# Comparison of Online Survey Recruitment Platforms for Hard-to-Reach Pregnant Smoking Populations: Feasibility Study

**DOI:** 10.2196/resprot.8071

**Published:** 2018-04-16

**Authors:** Jose Luis Ibarra, Jessica Marie Agas, Melissa Lee, Julia Lily Pan, Alison Meredith Buttenheim

**Affiliations:** ^1^ Department of Family and Community Health University of Pennsylvania Philadelphia, PA United States

**Keywords:** socioeconomic status, smoking, nicotine, cognitive bias, Web-based methods, crowdsourcing, delay discounting, vulnerable populations

## Abstract

**Background:**

Recruiting hard-to-reach populations for health research is challenging. Web-based platforms offer one way to recruit specific samples for research purposes, but little is known about the feasibility of online recruitment and the representativeness and comparability of samples recruited through different Web-based platforms.

**Objective:**

The objectives of this study were to determine the feasibility of recruiting a hard-to-reach population (pregnant smokers) using 4 different Web-based platforms and to compare participants recruited through each platform.

**Methods:**

A screener and survey were distributed online through Qualtrics Panel, Soapbox Sample, Reddit, and Amazon Mechanical Turk (mTurk). Descriptive statistics were used to summarize results of each recruitment platform, including eligibility yield, quality yield, income, race, age, and gestational age.

**Results:**

Of the 3847 participants screened for eligibility across all 4 Web-based platforms, 535 were eligible and 308 completed the survey. Amazon mTurk yielded the fewest completed responses (n=9), 100% (9/9) of which passed several quality metrics verifying pregnancy and smoking status. Qualtrics Panel yielded 14 completed responses, 86% (12/14) of which passed the quality screening. Soapbox Sample produced 107 completed surveys, 67% (72/107) of which were found to be quality responses. Advertising through Reddit produced the highest completion rate (n=178), but only 29.2% (52/178) of those surveys passed the quality metrics. We found significant differences in eligibility yield, quality yield, age, number of previous pregnancies, age of smoking initiation, current smokers, race, education, and income (*P*<.001).

**Conclusions:**

Although each platform successfully recruited pregnant smokers, results varied in quality, cost, and percentage of complete responses. Moving forward, investigators should pay careful attention to the percentage yield and cost of online recruitment platforms to maximize internal and external validity.

## Introduction

### Background

Smoking while pregnant is the leading cause of preventable infant morbidity and mortality, as well as pregnancy complications [1-6]. More than a quarter of women aged from 12 to 44 years in their first month of pregnancy report active cigarette smoking—and this prevalence is no lower than the rate among nonpregnant women aged 12 to 44 who are not pregnant [7]. Effective smoking cessation programs targeted at pregnant women can substantially reduce maternal and infant health outcomes, including infant mortality [8,9]. Although pregnant women who smoke have a higher quit rate during their pregnancy than any other time in their lives [10,11], only one-third of women are able to remain abstinent [12]. The high burden of disease associated with smoking during pregnancy coupled with the critical window of opportunity for cessation interventions generates substantial interest in research on pregnant smokers [13].

Recruiting a representative sample of pregnant smokers for descriptive or intervention studies is challenging [14]. Pregnant smokers may be reluctant to disclose smoking status due to social stigma (particularly in a clinical setting), making self-reported smoking status an error-prone measure. Furthermore, biologically confirmed smoking status (eg, cotinine testing) is expensive [15,16]. While in-person recruiting in prenatal care settings has been the norm in research to date, new opportunities have emerged to leverage social media and crowdsourcing to more easily recruit pregnant smokers as research subjects. Crowdsourcing platforms are Web-based marketplaces that allow researchers to post research tasks (such as survey completion) that interested subjects can complete for pay. Crowdsourcing offers an easy way to quickly recruit a large sample of study respondents that is more diverse (geographically and sociodemographically) than the typical college student young adult sample or an in-clinic patient sample [17]. Crowdsourcing platforms such as Amazon’s Mechanical Turk (mTurk), Soapbox Sample, and Qualtrics Panel Data draw a broad demographic of workers that can meet even very specific and targeted inclusion criteria [18,19]. Compensation for research task completion through crowdsourcing platforms is typically lower than in-person research participation with substantially lower research staffing costs, making crowdsourcing an appealing option for maximizing limited research budgets [14]. To date, Amazon’s mTurk has been the largest and best-known crowdsourcing platform due to low costs, flexibility, anonymity of workers, and, for researchers, the demographic diversity of the worker pool [20]. Social media is another online recruitment platform that extends researchers’ reach beyond the limitations of in-person recruitment. About 74% of the 2015 US population had Internet access and more than half of that population used at least one form of social media [21,22]. Social media allows for precise targeting of messages (including invitations to participate in research) to specific demographic profiles or interests. For example, the social media platform Reddit comprises many smaller interest groups called “subreddits” where members view other members’ posts, news, images, and media links. Advertisers, including researchers, can place ads on specific subreddits, such as a pregnancy or smoking cessation subreddit, to reach the desired target audience.

### Objectives

This study compares the characteristics of a sample of pregnant smokers (a small and temporally defined population) recruited through 4 Web-based platforms (3 crowdsourcing platforms and 1 social media site), then describes the feasibility of each platform and the cost per completed survey.

## Methods

### Study Design

We sought a sample of pregnant smokers aged 18 years and older living in the United States for a cross-sectional survey-based study of decision-making styles and preferences for incentive-based smoking cessation programs during pregnancy. The study was approved by the institutional review board of the University of Pennsylvania. Study respondents were first asked to complete a 6-question screener created on the Qualtrics Web-based survey platform to determine eligibility. Eligible respondents who provided informed consent then completed a 93-question survey about pregnancy history, smoking history, decision-making style, and smoking cessation program preferences (see Multimedia Appendix 1 for a sample of selected questions). Participants who did not consent were not allowed to continue onto the second survey. The recruitment period ran from July 6 to July 27, 2016. In total, 308 eligible respondents completed the survey. We evaluated platforms based off of two yields: eligibility yield, defined as the percentage of participants who met the inclusion criteria out of the number of total number of respondents, and quality yield, defined as the percentage of eligible participants who correctly and appropriately answered attention and quality checks embedded throughout the survey.

### Recruitment Platforms

Table 1 describes the recruitment platforms we used and their forms of recruitment flow, general cost, and options for researchers. We chose the platforms Amazon mTurk, Soapbox Sample, Qualtrics Panel, and Reddit due to their ease of use, relatively low cost, and the estimated number of respondents.

**Table 1:** Recruitment flow, cost for researcher, and options for targeting recruits by recruitment platform used, 2017.

Recruiting channel	Options for targeting recruits	Cost for researcher	Incentives for respondent
Amazon Mechanical Turk	None	Pay per completed task	US $0.01-US $0.02 for screening survey, US $0.10 for completion, up to US $0.70 based on quality
Soapbox Sample	Targeted based on demographics and interests	Pay minimum fee plus per completed survey	2000 points (US $2.00 equivalent)
Qualtrics Panel	Targeted based on demographics and interests	Pay minimum fee plus per completed survey	Paid by Qualtrics Panel
Reddit	Targeted based on interests	Pay when ads clicked or shown	US $10 e-gift card

#### Amazon Mechanical Turk

We used the third-party service TurkPrime (free to academic researchers) to anonymize respondents and to restrict survey dissemination to eligible and experienced mTurk workers. Participation was limited to those in the United States and those with a 95% approval rating after having completed more than 5000 human intelligence tasks (HITs in mTurk parlance) to maximize data quality. TurkPrime also batch-released the survey to ensure maximum visibility of the HIT. We varied the wording of the HIT titles (more vs less specific about survey content) to maximize participation. The screener survey initially paid US $0.01, but this was increased to US $0.02 to attract more respondents. The main survey paid US $0.10 with a US $0.70 quality bonus.

#### Soapbox Sample

We contacted Soapbox Sample for a price quote via telephone, and they provided an estimate of US $23 per completed survey for 75 to 100 respondents, with a minimum payment of US $500 after we asked to limit the sample to pregnant smokers in the United States. At no additional cost, Soapbox assigned a project manager who oversaw the number of eligible and completed surveys each day. Soapbox rewarded participants for completing the survey in points that could later be cashed in for gift cards from various retailers. Participants received 2000 points (US $2.00 equivalent) for this survey.

#### Qualtrics Panel

Qualtrics Panel is a subdivision of Qualtrics, a private research software company specializing in Web-based data collection that partners with over 20 Web-based panel providers to supply diverse, quality respondents. We contacted Qualtrics Panel for a quote via email and they provided an initial estimate price of US $20 per completed survey for 50 eligible respondents (pregnant women in the United States who smoke). However, they could not guarantee that the target sample size of 50 respondents would be met within their existing panels. The Qualtrics project manager noted that pregnancy is a “moving target,” in addition to the difficulty of Web assessment and underreporting of smoking status due to social stigma. The manager suggested pushing the survey through all platforms of their crowdsourcing platform, charging US $10 per survey completion with a minimum payment of US $500. This price included a project manager, who added embedded data into the survey for quality assurance and monitored attention checks. Participants were paid directly through Qualtrics.

#### Reddit

We first identified 4 Reddit subreddits of which pregnant smokers might be members. We initially planned to post a link to our screener survey directly to the most promising subreddits (eg, r/BabyBumps) but were informed by moderators that this type of survey or research promotion did not comply with subreddit guidelines. We quickly discovered that Reddit has an inexpensive and flexible auction-based system for placing advertisements. We ran several advertisements on promising Reddit subreddits pertaining to smoking cessation and pregnancy, including r/BabyBumps, r/TwoXChromosomes, r/stopsmoking, and r/Parenting. We experimented with various formats, text, and images across our different ad campaigns. All advertisements provided a link to the Qualtrics screener survey. We also varied our bid price per 1000 impressions to maximize our advertising budget. Eligible respondents who completed the main survey received a US $10 e-gift cards through GiftBit, a Web-based gift card service.

### Attention Checks and Quality Screens

As is typical in Web-based survey research, we employed multiple attention checks and quality screens in our surveys [23]. Attention checks confirmed that Web-based survey respondents were reading questions carefully and thoroughly. Quality screens attempted to confirm self-reported pregnancy and smoking status and confirmed that respondents spent an adequate amount of time completing the survey and were not simply checking response boxes as rapidly as possible (eg, selecting the same column repeatedly in a grid). Qualtrics Panel suggested using one-third of the median time to complete the survey as the cut-off point to determine whether respondents rushed through the survey, so we applied this criterion to every survey platform as a part of the quality screens. By platform, 0% (0/9) of respondents in mTurk, 13% (1/8) of respondents in Qualtrics Panel, 28.9% (31/107) of respondents in Soapbox Sample, and 16.9% (30/178) of respondents in Reddit did not pass the time-quality screens.

To confirm pregnancy status, quality screens checked for consistent self-reported gestational age, last menstrual period, estimated due date, and reports of real vs sham pregnancy symptoms. Quality screens for smoking status included knowing the number of cigarettes in a pack, experience of head rush when smoking (not typical for a regular smoker), and consistent reporting of smoking intensity. Additional quality screens included flagging when a respondent provided the same answers in a matrix of questions (ie, clicked answers in a straight vertical line down the page).

### Analysis

Completed eligibility screens and surveys from each recruitment platform were exported from Qualtrics to STATA v 14.2 (StataCorp, College Station, TX) for analysis. Descriptive statistics (mean and 95% CIs or proportions) were calculated for the completed sample by platform for the following measures: age, race, education, income, current smoking status (currently smoking in pregnancy or quit since beginning of this pregnancy), gestational age, and number of previous pregnancies. To analyze the descriptive statistics, we performed chi-square contingency test, analysis of variance, Kruskal-Wallis, and Fisher exact test. Cost data were compiled from invoices and receipts for subject payment and HIT management services (mTurk), gift cards (GiftBit for Reddit respondents), platform payments (Qualtrics Panel Data and Soapbox Sample), and Reddit ad purchases. Cost per completed survey was calculated as total costs per platform divided by number of completed, quality surveys. Eligibility yield was calculated by dividing the number of respondents who met the inclusion criteria by the number of total respondents per platform, whereas quality yield was calculated by dividing the number of quality surveys (number of completed surveys that pass the pregnancy screening, smoking screening, attention checks, quality checks, and email checks) by the total number of completed surveys per platform.

## Results

### Recruitment Outcomes

**Figure 1:**
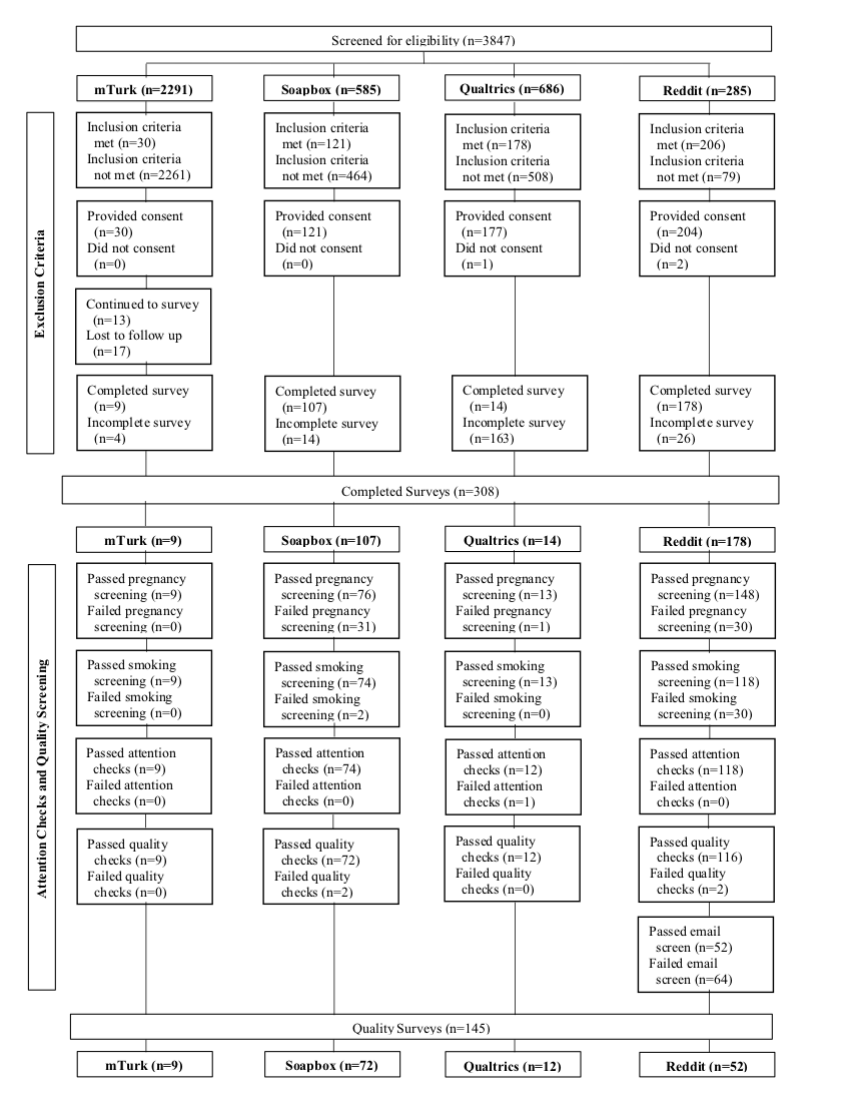
Recruitment outcomes at each stage of the recruitment process by platform.

Figure 1 presents recruitment outcomes at each stage of the recruitment process by platform. All platforms could identify pregnant women who smoke and who completed the study with sufficient quality, but the yields of quality surveys from total screen for eligibility varied considerably. We observed significant differences in eligibility yield, quality yield, age, number of previous pregnancies, age of smoking initiation, current smokers, race, education, and income (*P*<.001).

mTurk collected a total of 30 eligible respondents out of a total 2291 sampled (1.31% eligible). Of those eligible respondents, 17 failed to complete the trial survey (nonscreener portion of the overall survey) and are therefore considered lost to follow-up due to the partitioning of the survey into a screener survey and the trial survey to maximize the quality of the surveys, and 4 produced incomplete surveys, leaving a total of 9 completed surveys. After running the attention and quality screens on the completed mTurk surveys, 0 failed the pregnancy or smoking screener or the attention or quality screens for a quality yield of 100%.

Soapbox produced 20.7% eligible (121/585) respondents out of the total sampled respondents. Of those eligible, 14 produced incomplete surveys, leaving a total of 107 completed surveys. In total, 31 surveys failed the pregnancy check, 2 failed the smoking check, 0 failed the attention checks, and 2 failed quality screens for an overall yield of 60%.

Qualtrics collected a total of 25.9% (178/686) eligible respondents out of the total sampled respondents. Of those eligible, 1 did not provide consent and 163 produced incomplete surveys, leaving a total of 14 completed surveys. One survey failed the pregnancy check, 0 failed the smoking check, 1 failed the attention checks, and 0 failed quality screens for an overall yield of 7%.

Reddit collected a total of 72.3% (206/285) eligible respondents out of the total sampled respondents. Of those eligible, 2 did not provide consent and 26 produced incomplete surveys, leaving a total of 178 completed surveys. In total, 30 surveys failed the pregnancy check, 30 failed the smoking check, 0 failed the attention checks, and 2 failed quality screens for an overall yield of 65%.

Interestingly, the amount of surveys lost between each stage of checks varied across platforms. Although we received 177 eligible respondents in Qualtrics, only 14 (92.1%) completed the survey. mTurk’s respondents produced a similar pattern with 4 out of 13 (69%) incomplete surveys. Respondents from Reddit or Soapbox completed the survey more often than respondents from Qualtrics or mTurk, with Reddit’s incompletion rates at 12.8% (26 incomplete surveys out of 204 eligible surveys) and Soapbox’s at 11.6% (14 incomplete surveys out of 121 eligible surveys). Respondents in mTurk produced higher-quality responses than any other platform, with 0% of respondents failing any of the quality or attention screens. Similar to those recruited through mTurk, respondents from Qualtrics produced high-quality surveys. Out of 14 overall completed surveys, 1 survey failed the pregnancy screening and another survey failed the quality screens. Nearly 29% (one-third) of respondents in Soapbox failed the pregnancy screening, the highest failure rate of the pregnancy screenings across platform. However, aside from this, few others failed any other screenings: 2 failed the smoking screener and 2 failed the quality screens. Reddit had the most number of surveys that failed screeners. In total, 30 surveys failed the pregnancy screening and 30 others failed the smoking screener. Most respondents passed the attention checks and the quality screens. Surprisingly, all respondents passed the attention checks, and only 5 out of 208 total respondents did not pass the quality screen.

During survey collection, we noticed a sudden spike in the number of responses we received via Reddit. The timestamp on many of these responses occurred between midnight and 8 AM. The emails affiliated with them contained domain names from outside the United States—mostly from Eastern Europe—in spite of the fact that the Reddit ads had been geographically specified to target users in the United States. Furthermore, a pattern emerged in the domains of the emails we received from Reddit users, alternating between @me.com, @hotmail.com, and @gmail.com within a relatively short time frame. This series of events led us to believe someone disseminated our survey on the Internet as an easy opportunity to make money. Consequently, we manually combed through the email addresses to check for any repetitious email addresses and suspicious email domains. After closing our survey and ending the Reddit advertisement campaigns, we received a few emails from Reddit users claiming they had completed our survey but had not received payment. Because the 3 users who reached out to our team via Reddit had emails that were similar in structure, we sought to confirm their pregnancy status by asking her due date, last menstrual period, and number of weeks pregnant at the time of survey completion. After we received each response, we compared the information given in the email with the data collected from the survey responses and then sent the payment. Therefore, this decreased Reddit’s quality yield from 65% to 29%.

### Sample Characteristics

More than 50% of the total sample identified as white. Over half of all respondents have at least some college education. Most of the respondents had an annual family income of US $35,000 to US $74,999. Almost three-fourths of the respondents reported still smoking at the time of survey administration. As seen in Table 2, demographics varied widely across platforms. A substantial variation in the proportion of currently smoking respondents (vs recently quit during this pregnancy) existed: from 8% of Qualtrics respondents to 88% of Reddit respondents.

**Table 2:** Eligibility and quality yield, pregnancy, smoking, and demographic information by total and individual recruitment platforms, 2017.

Platform characteristics		Total (n=3847)	mTurk (n=2291)	Soapbox (n=585)	Qualtrics (n=686)	Reddit (n=285)	*P* value
Proportion eligible^a^, n (%)		535 (13.91)	30 (1.31)	121 (20.7)	178 (25.9)	206 (72.3)	<.001
Proportion of eligible deemed quality^a^, n (%)		145 (47.1)	9 (100)	72 (67.3)	12 (86)	52 (29.2)	<.001
Age^b^ in years, mean (SD)		28 (6.3)	33 (4.4)	30 (6.3)	29 (7.5)	24 (4.1)	<.001
Gestational age^b^ in weeks, mean (SD)		20 (12.4)	11 (13.8)	20 (13.0)	22 (9.4)	20 (11.6)	.21
Number of previous pregnancies^b^, mean (SD)		1 (1.3)	1 (0.7)	2 (1.6)	1 (1.0)	0.3 (0.7)	<.001
Initial age of smoking^c^, median (years)		18 (3.2)	17 (3.5)	17 (3.5)	17 (2.7)	19 (2.5)	.001
Current smokers^a^, n (%)		103 (71.0)	6 (67)	50 (69)	1 (8)	46 (89)	<.001
**Race^d^** **, n (%)**							
	White	82 (56.6)	8 (89)	53 (74)	6 (50)	15 (29)	<.001
	Black	28 (19.3)	0 (0)	11 (15)	3 (25)	14 (27)	
	Asian	10 (6.9)	0 (0)	4 (6)	1 (8)	5 (10)	
	Native American	8 (5.5)	1 (11)	1 (1)	2 (17)	4 (8)	
	Native Hawaiian	6 (4.1)	0 (0)	2 (3)	0 (0)	4 (8)	
	Prefer not to answer	11 (7.6)	0 (0)	1 (1)	0 (0)	10 (19)	
**Hispanic^d^** **, n (%)**							
	Non-Hispanic	119 (82.1)	8 (89)	60 (83)	10 (83)	41 (79)	.008
	Hispanic	18 (12.4)	1 (11)	12 (17)	1 (17)	3 (6)	
	Prefer not to answer	8 (5.5)	0 (0)	0 (0)	0 (0)	8 (15)	
**Education^a^** **, n (%)**							
	<High school	4 (2.8)	0 (0)	2 (3)	0 (0)	2 (4)	.004
	High school/ General Equivalency Diploma	47 (32.4)	0 (0)	16 (22)	5 (42)	26 (50)	
	Some college	31 (21.4)	2 (22)	15 (21)	2 (17)	12 (23)	
	Associate's	17 (11.7)	1 (11)	11 (15)	0 (0)	5 (10)	
	Bachelor's	33 (22.8)	6 (67)	19 (26)	3 (25)	5 (10)	
	>16 years	11 (7.6)	0 (0)	9 (13)	2 (17)	0 (0)	
	Prefer not to answer	2 (1.4)	0 (0)	0 (0)	0 (0)	2 (4)	
**Income^a^** **(US $), n (%)**							
	<$10,000	18 (12.4)	0 (0)	7 (10)	1 (8)	10 (19)	.004
	$10,000-$14,999	6 (4.1)	1 (11)	3 (4)	0 (0)	2 (4)	
	$15,000-$19,999	15 (10.3)	1 (11)	6 (8)	1 (8)	7 (13)	
	$20,000-$24,999	21 (14.5)	1 (11)	5 (7)	0 (0)	15 (29)	
	$25,000-$34,999	15 (10.3)	0 (0)	5 (7)	4 (33)	6 (12)	
	$35,000-$49,999	24 (16.6)	4 (44)	13 (18)	1 (8)	6 (12)	
	$50,000-$74,999	21 (14.5)	1 (11)	15 (21)	2 (17)	3 (6)	
	≥$75,000	19 (13.1)	1 (11)	14 (19)	3 (25)	1 (2)	
	Prefer not to answer	6 (4.1)	0 (0)	4 (6)	0 (0)	2 (4)	

^a^Comparison across platforms by chi-square contingency test.

^b^Comparison across platforms by analysis of variance.

^c^Comparison across platforms by Kruskal-Wallis test.

^d^Comparison across platforms by Fisher exact test.

**Figure 2:**
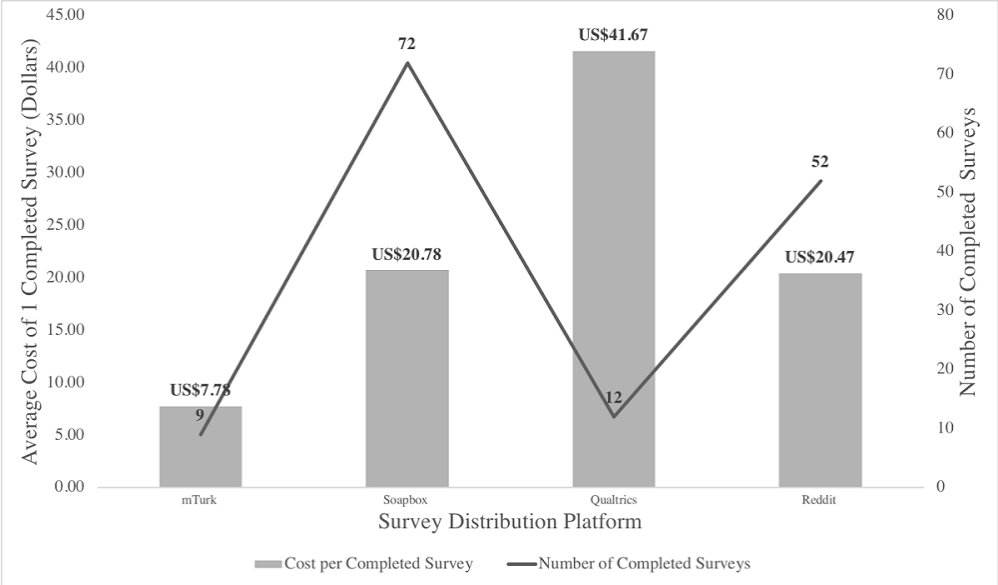
Cost per completed survey compared with the number of completed quality surveys by platform, 2017.

#### Cost per Completed Survey

Cost per completed survey and total number of completed surveys are shown in Figure 2. By far the cheapest method for distributing surveys, mTurk had an average cost per completed quality survey of US $7.78 (including the cost of completed screener surveys.) However, there seems to be a trade-off between average cost and survey completion. This platform yielded some of the fewest completed surveys.

Soapbox Sample placed a minimum fee of US $500 and priced each survey at US $24.93; after the amount of surveys we received exceeded US $500, each additional survey cost US $24.93. Because Soapbox produced a relatively high amount of low-quality surveys, the Web-based recruitment company only charged us for 60 high-quality surveys. For a total of US $1495.80 and 72 completed overall surveys, the price per completed survey was US $20.78.

Similar to Soapbox Sample, Qualtrics Panel placed a minimum fee of US $500 until the cost of completed responses exceeded US $500 (at US $10 per completed survey). Qualtrics Panel was unable to guarantee a minimum number of responses given our narrow inclusion criteria. With only 12 completed surveys from Qualtrics Panel, each completed survey cost US $41.67.

For the Reddit platform, we spent US $122.67 on Reddit ads, running a total of 9 ad campaigns on 4 subreddits that received a total of 146,885 impressions and 350 clicks. We received 178 completed responses, 95 of which received a US $10 e-gift card using Giftbit. Of the gift cards sent, 9 respondents accepted the gift but never used their reward. These respondents allowed the gift to expire and 1 respondent even canceled his or her gift. Therefore, we utilized US $850 of the US $950 spent on rewards. The average cost per completed survey was US $20.37.

## Discussion

### Principal Findings

In this explanatory analysis, we compared the yield from and cost of four Web-based survey respondent platforms for carrying out a cross-sectional study of a hard-to-reach population: pregnant smokers. The quantity, quality, and cost of completed surveys varied widely across platforms. Note that without optimized or standardized recruitment methods, we will have variation in yields by definition.

Soapbox and Qualtrics Panel, two similar services offering existing panels of survey respondents, produced very different yields, with the Soapbox producing more eligible surveys by a factor of 6 (Soapbox, n=72; Qualtrics Panel, n=12). The 2 platforms produced similar quality yields: 67% of Soapbox surveys and 86% of Qualtrics Panel passed the quality screens. Both companies described the challenge of recruiting pregnant smokers to complete our surveys upfront. Although Qualtrics produced a low eligible yield (26%), it produced the second-highest quality percentage of 86%. In contrast, Soapbox recruited a higher number of respondents than Qualtrics Panel could, but only 67% of Soapbox surveys were able to pass the quality screens. Going forward, we would be more likely to use Soapbox than Qualtrics Panel, given the higher yield.

Amazon’s mTurk platform, which claims to have over 500,000 workers, produced a very low eligible yield (n=9) but the highest-quality surveys (100%). We attribute the high-quality yield to using only “mTurk Masters” who had a 95% approval rating. However, use of this selective qualification could similarly have limited the number of eligible participants, attributing to our low eligible yields. Loss-to-follow up from our screener to the main survey contributed to the low yield; going forward, we will likely combine the screener into the main survey, pay a smaller fee for the screener portion, and a quality bonus for eligible completers.

Placing ads on Reddit subreddits initially appeared a promising way to drive eligible respondents to our survey. The ads we placed produced 178 completed surveys with an eligibility yield of 72%. However, its proportion of quality surveys was the lowest, with a quality yield of 29%. We were also subject to an unfortunate “hack” of the survey. This “hack” seriously diminished the credibility of the survey results derived from this platform. Going forward, we would be unlikely to use Reddit to disseminate surveys.

For cross-sectional observational studies such as our survey, the ability to generalize results from the sample to a broader population is crucial [24]. We noted distinct sociodemographic profiles across our 4 platforms, with more variability in Reddit and mTurk samples and less in the Qualtrics and Soapbox samples. This is not surprising given, again, the very narrow inclusion criteria for our sample. Reddit and Soapbox contributed the most demographic variability in terms of gathering responses from people in various races, education levels, and income brackets. This cross-platform variability appears to somewhat alleviate the threats to external validity that come with collecting information solely through one platform. However, the benefits of multiple platform recruiting do come at a significant cost—multiple platform recruiting multiplies the complexity and monetary expenses of running a study.

### Limitations

We note 4 important limitations of our explanatory study. We explored only 4 of the various online recruitment platforms that could be leveraged for participant recruitment. At the time when the study was conducted, Reddit identified 12,927,467 active users. Platforms such as Facebook or Twitter may have been able to be used because of their wider user base, with Facebook boasting 1,712,000,000 users and Twitter 313,000,000 users as of the second quarter in 2016. However, Facebook’s inability to identify pregnant and smoking women in its advertising options prevented its usage in this study. Although Twitter does allow users to produce ads much like Reddit, Twitter’s reach also depends on the sender’s popularity. That is, many Twitter users must first “follow” the advertiser in order to see the advertiser’s ads. Another limitation of the study is consistency across platforms. For mTurk and Qualtrics, sample size was relatively limited. As with all studies utilizing online recruitment methods, our study relied on self-reported information. This presents the possibility that not all responses are completely accurate. A third limitation is that the method in which we recruited through Reddit may have yielded inaccurate responses. The mentions of “pregnancy” and “smoking” in our ads may have primed potential participants. This also may have been the reason for the “hack” that we experienced toward the end of the advertising campaign. Next, we recognize that we could not verify smoking status via Web. Although we attempted to design our quality screens by asking about the number of cigarettes in a pack and their preferred brand, we realize this is not a proven method of verifying smoking status. This is usually not an issue faced during in-person recruitment. For most in-person studies, smoking verification methods such as urine cotinine tests are more reliable and can be performed in the setting of a clinic. Lastly, it is important to address the intrinsic differences in the platforms that could have led to variation. First, the methods to target pregnant smokers vary by platform such that some, such as Reddit, are based off of subscriptions and readership while others, such as mTurk, are based off of demographic probability. Therefore, platforms that allow for customization and targeting might lead to a higher percentage of eligible participants than platforms that do not allow for customization. Second, given that the method of incentivizing differs across platforms, users of one platform may be more willing to complete the survey than users of another platform. However, to ensure generalizability, we attempted to use each platform as a typical research would and therefore ensured that the incentive participants received in each platform was similar to those of past researchers in the same platform. Finally, although we do not find it to be a limitation, we note that the difference in methods between mTurk and the other platforms may raise concerns. Turk Prime’s specific ability to only administer HITs to specific mTurk workers based on their anonymous ID meant that mTurk was the only platform where identifying information would not be collected but researchers could still follow up to respondents. In contrast, the Reddit platform required contact information to use as a screener. Therefore, the addition of the screener for mTurk’s platform is more of an asset than a limitation to our study.

More broadly speaking, there are limitations in the use of online recruitment when compared with in-person clinical recruitment. Online recruitment methods are limited by demographic representation, biases, and uncertainties. By nature, samples recruited through Web-based methods are not representative of the broader target population [25]. For example, racial and ethnic disparities exist in the accessibility and frequency of computer use in the United States. However, these are minimized when analyzing Internet access via mobile devices [26]. Similarly, those who participate in studies hosted on mTurk tend to be younger, more liberal, and more familiar with Web-based technology [26-28]. Nevertheless, although mTurk is less representative than Web-based panel services or national probability samples, it may provide a more representative sample of the United States than traditional in-person sampling methods [29]. Online recruitment may also yield lower-quality data as this paper has shown. Accountability and validity are generally more difficult to enforce in online research. Web-based studies tend to rely on self-report, and subjects can more easily provide responses that do not reflect their actual beliefs, values, or behavior. On mTurk, “spammers” and “bots” capitalize on this and find ways to receive rewards offered by a study without successfully completing the intended task [18,30] with obvious adverse consequences for the data validity. Inattention and lack of intrinsic motivation may lead to superficial responses and higher attrition rates, although this can be mitigated somewhat by attention checks—supplementary questions and tasks that determine whether a participant is fully paying attention [18]. While the distance between researcher and subject may reduce social desirability bias, Web-based research is not immune from it [27].

### Comparison With Prior Work

Our findings are not consistent with recent studies looking at online recruitment yields, cost, and representativeness. Other studies that have focused on mTurk as a method to recruit participants have concluded that the Web-based service is relatively inexpensive and efficient [31]. Select studies have further suggested that small payment amounts do not appear to significantly detract from quality [20]. Although we have confirmed that mTurk is indeed inexpensive in our study, it may not, however, have been the most cost-effective recruitment method for our purposes. The number of quality responses that we obtained through our mTurk recruitment efforts was smaller than desired. In part, this may have been due to the specificity of our selection criteria. Indeed, research has shown that mTurk samples tend to be more diverse and thus, more representative of the general population than other Web-based and in-person recruitment methods [29]. Consequently, mTurk may be a more attractive method of recruitment for studies that have less stringent selection criteria than ours.

However, our survey confirmed recent literature regarding online recruitment for hard-to-reach populations. In a study conducted by Martinez et al, the researchers acquired tens of thousands of impressions on different Websites such as Facebook and Craigslist and mobile phone apps such as Instagram, Grindr, and Jack’d to recruit HIV-positive gay Latinos [32]. Similarly, our study used various platforms (subreddits) within the large social media Reddit platform to push our survey to populations of interest. After reaching over 100,000 viewers, we received about 200 completed surveys, many of which were of dubious quality or validity. Given that Admon et al used Facebook to recruit a large robust sample of pregnant women through advertisements at very low costs, future research is needed to compare these crowdsourcing platforms and others with more social media sites such as Facebook, Twitter, and Instagram [33]. Furthermore, future studies should use an in-person sample as a baseline to compare the Web-based platforms and determine efficacy.

### Conclusions

This explanatory study confirmed significant variability in recruitment success, quality, and cost across multiple Web-based survey research platforms and social media recruitment strategies. With one exception (mTurk), we observed an inverse relationship between cost per completed survey and number of surveys completed; sample characteristics also varied by platform. We procured higher quality samples from portals that prescreened respondents for us (Soapbox and Qualtrics Panel) vs platforms that draw from a larger pool of potential respondents (mTurk and Reddit). The results of these recruitment efforts suggest that it remains challenging to strike an optimal balance between quality and quantity when recruiting hard-to-reach subjects through Web-based platforms.

## Appendix

Multimedia Appendix 1 Sample of questions eligible respondents answered from survey.

## Abbreviations

HIT: human intelligence task
mTurk: Amazon Mechanical Turk
