# Supplementary material for: Comparison of Online Survey Recruitment Platforms for Hard-to-Reach Pregnant Smoking Populations: Feasibility Study
Source: JMIR Res Protoc. 2018 Apr 16;7(4):e101. doi: 10.2196/resprot.8071 (PMC5928332; doi:10.2196/resprot.8071)
Supplement: Multimedia Appendix 1 [file resprot_v7i4e101_app1.pdf]

Before this pregnancy, how many times have you given birth?

____

At what age did you first become pregnant?

____

As of today, how many weeks pregnant are you?

____

When was the first day of your last menstrual period? Please type your answer in the following format: mm/dd/yyyy

____

What brand of cigarettes do you usually smoke?

____

About how many cigarettes do you smoke a day?

____

How many cigarettes are in a standard box of cigarettes?

____

When is your due date? Please type your answer in the following format: mm/dd/yyyy

____

Do you pack your cigarettes before lighting them?

- ○ Yes
- ○ No

Have you experienced swelling anywhere on your body during this pregnancy?

- ○ Yes
- ○ No

When was your last prenatal visit? Please type your answer in the following format: mm/dd/yyyy

____

Have you had an ultrasound (sonogram) during this pregnancy?

- ○ Yes
- ○ No
- ○ Not yet

Have you had a glucose tolerance test during this pregnancy (where you drink the sweet drink)?

- ○ Yes
- ○ No
- ○ Not yet

Have you suffered from any of the following pregnancy symptoms? [Check all that apply]

- ☐ Pregnancy elbow
- ☐ Hearing loss
- ☐ None

Do you usually experience a head rush after smoking?

- ○ Yes
- ○ No

Were you prescribed zinc supplements during this pregnancy?

- ○ Yes
- ○ No

How much does a pack of your favorite cigarettes cost in your area?

____

How soon after waking do you smoke your first cigarette? (If you have recently quit smoking, think back to before you quit smoking.)

- ○ Within 5 minutes
- ○ 5-30 minutes
- ○ 31-60 minutes

Do you find it difficult to refrain from smoking in places where it is forbidden? e.g. Church, Library, etc. (If you have recently quit smoking, think back to before you quit smoking.)

- ○ Yes
- ○ No

Which cigarette would you hate to give up? (If you have recently quit smoking, think back to before you quit smoking.)

- ○ The first in the morning
- ○ Any other

Do you smoke more frequently in the morning? (If you have recently quit smoking, think back to before you quit smoking.)

- Yes
- No

Do you smoke even if you are sick in bed most of the day? (If you have recently quit smoking, think back to before you quit smoking.)

- Yes
- No

Twice in this survey, you were asked to rate the likelihood of certain scenarios occurring. Each time, the scenarios involved the likelihood of events occurring to you or another participant in this study. In what order did these questions appear in this survey?

- Another participant, then me
- Me, then another participant
- Me, then me again
- Another participant, then another participant again
